# The Curvilinear Relationships Between Relational Embeddedness and Dynamic Capabilities: The Mediating Effect of Ambidextrous Learning

**DOI:** 10.3389/fpsyg.2022.830377

**Published:** 2022-04-07

**Authors:** Yina Zhang, Jiancheng Long, Wu Zhao

**Affiliations:** ^1^School of Economics and Management, Xidian University, Xi'an, China; ^2^School of Marxism, Xidian University, Xi'an, China

**Keywords:** relational embeddedness, exploratory learning, exploitative learning, dynamic capability, BOP

## Abstract

Dynamic capabilities are crucial to the survival and development of enterprises in the BOP (Base/Bottom of the Pyramid, hereinafter BOP) market. The research focuses on the double-edged sword impact of relational embeddedness on dynamic capabilities *via* ambidextrous learning, that is moderate embeddedness facilitates dynamic capabilities while overembeddedness inhibits them. Furthermore, this study investigates whether human capital moderates the relationships between relational embeddedness and ambidextrous learning. Selecting 264 samples for empirical research, firstly, the results show that the relational embeddedness in the BOP cooperation network has an inverted U-shaped influence on ambidextrous learning and dynamic capabilities. Secondly, exploratory learning and exploitative learning play a mediating role in relational embeddedness and dynamic capabilities. Thirdly, prior experience plays a positive moderating role in relational embeddedness and exploitative learning. The conclusions facilitate understanding the antecedents of dynamic capabilities and the black box of “embeddedness paradox,” and provide empirical evidence for adjusting the human capital of enterprises, enhancing the exploratory learning capability and exploitative learning capability, and coping with the overembeddedness effects.

## Introduction

For a long time, companies have paid more attention to the high-end market in the pyramid composed of wealthy groups and the middle class (Top of the Pyramid, hereinafter TOP), and ignored the BOP groups living in the vast underdeveloped areas, which account for more than 2/3 of the world's population (Nakata and Weidner, 2012; Yurdakul et al., 2017). BOP strategy proposed by Prahalad and Hart (2002) believes that the enormous low-income people that are rarely included by business encompass huge potential wealth. Only by absorbing BOP groups into the market economy, can enterprises generate profits while simultaneously alleviating poverty (Hart and London, 2005). Due to the weak infrastructure, low educated workforce, and informal governance mechanisms (Webb et al., 2010; World Bank Statistics, 2019; Sun et al., 2020), different participants need frequent and direct interaction to ensure the smooth progress of production and trading activities. As Hart (2015) argued that most BOP ventures and corporate initiatives over the past decades have either failed outright or dramatically underperformed against expectations at great cost. Being embedded in a BOP cooperation network (hereinafter BOP network) with specific relationships and structures established by non-traditional partners such as local government, non-governmental organizations (NGO), community organizations, and the poor themselves is critical to survival (Clarke and Fuller, 2010). Founding a diversified network relationship with non-traditional partners is a significant means for enterprises to deal with a dynamic environment (Døving and Gooderham, 2008). Concerning the particularity of the BOP market, enterprises call for adaptation to local norms and building on local conditions, and are also expected to radically change the context by introducing new products and services. Thus, how does relational embeddedness affect dynamic capabilities, managing their continuous adaptation to and shaping the environments to survive?

However, although scholars generally believe that an increase in uncertainty requires strong dynamic capabilities, including the capacities to explore, learn and adapt (Teece and Leih, 2016), there has still been criticized for offering only a limited understanding of how dynamic capabilities emerge and evolve (Peteraf et al., 2013). Existing research on dynamic capabilities has identified several antecedents, such as knowledge absorption capability (Saenz et al., 2014), relational management (Forkmann et al., 2016), relational learning (Smirnova et al., 2018), network resources (Alinaghian and Razmdoost, 2017), and relational embeddedness (Frasquet et al., 2018; Alinaghian et al., 2020). Dynamic capabilities beyond a single firm's boundaries are gaining more and more attention (Forkmann et al., 2018). Although the influence of relational embeddedness on firms' dynamic capabilities has been recognized by a plethora of research (e.g., Frasquet et al., 2018; Alinaghian et al., 2020), quantitative empirical evidence is still limited. Additionally, whereas previous studies often investigated the influence of relational embeddedness on innovation (Albis et al., 2021), firm performance (Mozumdar et al., 2019), firm growth (Bird and Zellweger, 2018; Zeevik et al., 2018), an increasing number of literature is now examining the impact of relational embeddedness on the dynamic capabilities to survive and grow in the face of uncertain environments (Frasquet et al., 2018; Alinaghian et al., 2020; Zhou et al., 2021). But these studies emphasized the positive effect of relational embeddedness (Zheng et al., 2011; Rodrigo-Alarcón et al., 2018; Ai and Peng, 2021; Zhou et al., 2021), and neglected the darkness of overembeddedness itself. Eriksson (2014) called for more comprehensive empirical research on dynamic capabilities. Second, an issue that the organizational learning and dynamic capabilities literature has addressed is that learning is a vital way for enterprises to build dynamic capabilities (Eisenhardt and Martin, 2000; Zollo and Winter, 2002; Pu and Soh, 2018). However, few scholars in the BOP market have noticed the relationship between knowledge, learning, and dynamic capabilities (Eisenhardt and Martin, 2000; Teece and Leih, 2016). In addition, a line of research has argued that relational embeddedness can also affect learning (Uzzi and Lancaster, 2003; Pu and Soh, 2018). We found few studies that connect the two. Noting this, investigating the possible mediating role of ambidextrous learning between relational embeddedness and dynamic capabilities has also become an important research direction because evidence of the mediating effects can prompt firms' attention to the issue of ambidextrous learning when improving dynamic capabilities. Taken together, the second goal of the current study is to investigate whether ambidextrous learning would positively mediate the relationship between relational embeddedness and dynamic capabilities. Finally, the effects of relational embeddedness on different themes of ambidextrous learning may vary, or even contradict, one another in their moderation by human capital (e.g., prior experience). This is because human capital may have a negative effect on ambidextrous learning by increasing cognitive inertia, and lock-in effects while promoting it by providing stronger cognitive capability, and communication capability. Considering this, the third goal of our study is to investigate whether and how prior experience, e.g., knowledge and resources from prior industry experience and BOP market experience, moderates the relationship between relational embeddedness and ambidextrous learning.

To close these gaps, we distribute the survey and collect 264 responses *via* a popular online platform in China named Credamo with a total of over 2.8 million registered samples, comprehensive coverage of all provincial administrative regions in China, and support for hundreds of user tags. For example, the questionnaires are accurately delivered according to the characteristics of the subjects such as gender, age, occupation, and region.

This paper tests its hypotheses by employing questionnaire survey data of different industries, where enterprises located in concentrated contiguous areas in the Qinba Mountains Shaanxi province. Adopting social embeddedness theory, dynamic capability theory, and ambidextrous learning theory to explore the influence of relational embeddedness and ambidextrous learning on the dynamic capabilities in the BOP context, as well as the moderating effect of prior experience. The contributions of this study are as follows: First, this study provides a new understanding of the dynamic capabilities' antecedents and the black box of the “embeddedness paradox” in the BOP context. The study empirically confirms the notion that the impact of relational embeddedness on dynamic capabilities is a curvilinear relationship. This is a complement to those of Rodrigo-Alarcón et al. (2018), and Ai and Peng (2021) who believe that relational capital may positively predict dynamic capabilities. Thus, our findings expand domains of the dynamic capabilities' antecedents and “embeddedness paradox.” Second, the study consolidates support for the dynamic capabilities through the perspective of social embeddedness and ambidextrous learning. It advances the literature of dynamic capabilities by showing that as a mediation device, ambidextrous learning may transform useable resources from relational embeddedness into dynamic capabilities, expanding the previous point of view (Aranda et al., 2017; Yuan et al., 2021). Finally, from the perspective of human capital, we explore the boundaries of the double-edged sword impact of relational embeddedness on ambidextrous learning. That is to say, under what context, moderate embeddedness brings positive effects and overembeddedness leads to negative effects. This study promotes understanding of the contextual factors that affect the inverted *U*-shaped relationship between relational embeddedness and ambidextrous learning and its contingency mechanism.

## Theoretical Basis

### Relational Embeddedness

The enterprise network theory agrees that in addition to its own resources, enterprises can also obtain key resources through various forms of connections with external entities. Various connections between enterprises can bring considerable relationship rents and competitive advantages to enterprises (Dyer and Hatch, 2006). Relational embeddedness mainly studies the problem of binary transaction relationships between network participants, that is, the degree of mutual trust and commitment between transaction parties (Gulati, 1999). Uzzi (1999) research found that embedded connections through three aspects (trust, high-quality information sharing, and joint problem-solving mechanisms) enable companies to obtain benefits such as reducing transaction costs, acquiring scarce resources, reducing environmental uncertainty, and promoting organizational learning. First, trust is the most important aspect of relational embeddedness (Garcia-Villaverde et al., 2018). Inter-organizational trust can be regarded as a resource, which can alleviate the speculative behavior that may be caused by uncertainty and dependence in transactions. Trust also generates flexibility in coping with situations of uncertainty (Nonino, 2013), and helps reduce the costs of market transactions (Czernek-Marszaek, 2020a). Second, an information-sharing mechanism provides support and guarantee for the transmission and flow of information and emotions between organizations. Information sharing means that both parties are willing to share tacit knowledge that goes beyond the public information in the market. Information exchange, trust, and cooperation between enterprises make it possible for enterprises to obtain corresponding external resources by means of the Internet (Inkpen and Tsang, 2005; Boso et al., 2013). Third, joint problem-solving refers to the sharing of responsibilities between related companies, coordinating with each other, and jointly solving the problems that arise as the relationship deepens (Heide and Miner, 1992). By solving problems together, partners establish a common habit and language system, which is more conducive to the transfer of complex knowledge blocks. The flexibility of attitudes and actions of the partners enables them to solve problems with the use of the limited resource (Czernek-Marszaek, 2020a).

The paradox of relational embeddedness has existed for a long time. Granovetter (1973) divided social network relationships into strong ties and weak ties in his representative work “The strength of weak ties.” He emphasized the role of weak connections in acquiring heterogeneous information and knowledge. Burt (1992) stated that weak ties are the advantage of corporate innovation. Weak connections are easy to enhance enterprises flexibility and achieve cross-organizational communication, and increase the breadth of knowledge. Weak ties can also greatly reduce the cost of acquiring knowledge. Hansen (1999) concluded that weak ties are good for searching and discovering useful knowledge while strong ties are good for transferring complex knowledge. Some scholars believed that strong ties are beneficial to the acquisition of corporate knowledge by enhancing trust and the improvement of innovation performance (Levin and Cross, 2005; Wang et al., 2020). Strong ties also can better regulate and restrict the behavior of partners, and promote learning and imitation between organizations (Tsai, 2009). However, too high a level of this embeddedness leads to the so-called “overembeddedness effect” bringing negative consequences for business activity. Czernek-Marszaek (2020b) identified possible negative consequences of social embeddedness for cooperation, such as lower adaptation abilities caused by adjusting to known partners, accusations of nepotism in cooperative relations, limiting the willingness to cooperate, and susceptibility to the opportunistic activities of a partner. The development and maintenance of strong ties can be associated with high costs in the form of time and resource allocation (Obukhova and Zhang, 2017). Enterprises may rely too much on their partners, creating a relationship lock-in risk (Uzzi, 1997; Rowley et al., 2000).

For small firms, like those in the BOP market, a network is more likely to be informal and consists of social links with individuals such as family, friends, and acquaintances. The connections within the BOP cooperation network are mostly highly personal and informal. London et al. (2010) proposed that relational embeddedness constructs a value chain or value network that adapts to BOP groups, and it can identify production and transaction constraints that restrict or hinder the resources and capabilities of low-income groups and release their value creation potential. Goyal et al. (2016) found that relational embeddedness enhances the socioeconomic impact and sustainability of the BOP market. Enterprises can only obtain high-quality knowledge and information by embedding it in the local network, improving operational efficiency, and accessing new markets (Sánchez and Ricart, 2010). While the positive effects of social embeddedness have been relatively more often discussed in the literature, the negative effects are rarely the subject of in-depth considerations (Mitrega and Zolkiewski, 2012). Meanwhile, relatively few empirical studies are available to verify this conjecture. To fill this gap, we test the curvilinear effect of relational embeddedness in the BOP market.

### Dynamic Capability

In his seminal paper, Teece et al. (1997, p. 515) defined dynamic capabilities as “the key role of strategic management in appropriately adapting, integrating, and reconfiguring internal and external organizational skills, resources, and functional competencies to match the requirements of a changing environment.” He found that dynamic capability is the ability to adapt to the external environment in nature. Meanwhile, the enterprises can not only passively adapt to the environment but also can change or even reshape the environment through corresponding activities (Teece, 2007). Teece (2007) divided it into sense, adaptation, and shaping capabilities from an environmental perspective. Environmental sensing capability means that enterprises can explore and obtain sources from the external environment, helping them seize market opportunities. Adaptation relates to routines of resource exploitation and deployment focusing on external changes, which can identify and tap more market opportunities and can quickly adapt to a volatile environment (Dixon et al., 2014). Adaptation is critical to the evolution and survival of enterprises. Shaping capability takes advantage of innovation opportunities and transforms them into innovation results consisting of a series of routines and processes. Enterprises can generate or cultivate a local supply chain by, for example, training and educating raw material producers, financial institutions, and/or local labor (Ausrød et al., 2017), which would shape the context and create a more suitable environment in turn.

Scholars now increasingly recognize that fostering dynamic capabilities often extends beyond a single firm's boundaries (Forkmann et al., 2018). For instance, When O'Reilly and Tushman (2008) investigated how to solve the innovator's dilemma, in order to build and cultivate dynamic capabilities, firms must scan, search, and explore across technology and market boundaries. Tran et al. (2019) proposed that new dynamic capabilities mature over time through the integration of operational capabilities to adapt to changes in the environment with external partners. Working jointly toward the goal may also involve changing this goal to shape the environment (Artto et al., 2016). Dynamic capabilities can help companies reshape the environment in a complex external environment, change the rules of the game, and enhance their competitive advantage. In the process of adapting to and shaping the external environment across the firm's boundaries, dynamic capabilities realize the survival and sustainable competitive advantage of the enterprise.

The aforementioned research mainly focuses on non-BOP areas, and the availability of external resources is an important basic assumption for them. When faced with the BOP scenario, this assumption will undergo a fundamental change. Ansari et al. (2012) pointed out that the market environment in the BOP region is significantly different from the mature market, and companies cannot directly obtain resources from the BOP market (Mair et al., 2012). To promote the success of BOP-oriented business activity, companies are eager to attract potential partners who have the resources needed for business development. Empirical studies have, however, found that business strategies that adapt to impoverished environments by leveraging local institutional strengths tend to outperform those grounded in the business conditions of developed economies (London and Hart, 2004). Therefore, the mechanism of the dynamic capabilities at the BOP may be different from that of the non-BOP market. Few studies have analyzed the dynamic capabilities at the BOP. The BOP offers a suitable context in which to explore the dilemma concerning the adaptation to and shaping of the context because firms are advised to adapt to their context and build on local conditions (Hart and London, 2005). Emphasizing the adaptation to local norms and/or negotiating mutually acceptable practices, enterprises should focus on the dynamic long-term engagement between a lead firm and their BOP producers (Ramachandran et al., 2012). Managers should adapt their BOP strategies to an industry environment (Zhu et al., 2019). Simultaneously, firm activities are also expected to radically change the context in which they operate by introducing new products and services (Prahalad and Hammond, 2002), improving the dynamic capabilities of the enterprises in the local area. Tashman and Marano (2009) claimed that base of the pyramid dynamic capabilities targeting firm value chain and the business environment involve resource integrating, transforming, acquiring, and shedding capabilities cooperated with grassroots communities, the people experiencing poverty, local government and enterprises, adapting to and shaping local entity and environment. Although prior research has made many valuable contributions, several important issues remain understudied. In the context of BOP, the generation mechanism of dynamic capabilities is still vague. Thus, there arises an urgent need to examine the effect mechanism of dynamic capabilities across a firm's boundaries in the BOP context.

## Hypothesis Development

### Relational Embeddedness and Dynamic Capability

Concerning the environmental characteristics of the BOP market, relational embeddedness is an important means to reduce transaction costs, eliminate environmental uncertainty, and obtain scarce resources in the BOP market (London et al., 2010). Mutual trust established by frequent interaction promotes the effectiveness of sharing information and advances the accuracy of information obtained (Mcevily and Marcus, 2005). Enterprises can integrate and deploy internal and external resources, and perceive changes in the external environment earlier and timely. As the degree of embeddedness deepens, partners share information more actively and voluntarily (Uzzi, 1997). They tend to provide more specific and implicit information, such as information on possible problems and opportunities foreseen, market and technological developments and trends, all of which can improve the accuracy of enterprises' expectations, enhancing their environmental sensing capabilities. In the BOP market, many important strategic resources cannot be obtained through market transactions (Seelos and Mair, 2007). Only by maintaining the information sharing of network members can enterprises obtain high-quality tacit knowledge, improve operational efficiency, and access new markets (Sánchez and Ricart, 2010; Sun et al., 2021). Mutual trust can not only help enterprises gain recognition and acceptance but also fill the institution gaps through social interaction and cooperation, ensuring the effective implementation of informal agreements (Ansari et al., 2012) and reducing BOP opportunistic behaviors (Reficco and Márquez, 2012). Fainshmidt and Frazier (2017) held that mutual trust shows stronger adaptability, stress resistance, and durability in a dynamic environment, and provides enterprises with more flexible strategic choices. Non-profit organizations and BOP groups in the BOP market can reduce costs in all aspects of raw material supply, manufacturing, circulation, and sales, and the participation of government departments and community organizations can effectively resolve transaction risks in the BOP market and improve transaction efficiency (Dahan et al., 2010). Solving problems together usually leads to joint action, making it easier to obtain local legitimacy. Then, they can acquire local scarce resources and skills, promoting the reconstruction of the corporate value chain and building business models. The efficiency of corporate environmental adaptation and shaping capabilities is improved.

On the contrary, excessive relational embeddedness brings a negative impact. Excessive trust easily leads to the reduction of cognitive effectiveness (Batjargal and Liu, 2004; Czernek-Marszaek and Czakon, 2016) and the illusion of control in the decision-making process of enterprises, thus underestimating the risk and quality of the enterprises in acquiring resources (Czernek-Marszaek, 2020b), which affects the perception of risks of enterprises and loses the ability of enterprises to flexibly respond to market changes. Excessive trust will also increase the risk of free-riding (Chowdhury et al., 2016). Excessive information sharing will cause the information to be locked and damage its dynamic capabilities. Its time consuming and resource-intensive will squeeze the possible ties with other network entities, inhibit the acquisition of non-redundant heterogeneous information and new opportunities from the external environment (Zhou et al., 2014), and reduce their adaptability to the external environment of the network (Burt, 1992). Path dependence caused by overembeddedness will restrict their thinking and flexibility in solving new problems, and limit the formation of their ability to solve problems independently. The resulting network inertia weakens the flexible response and processing capabilities of enterprises in the face of uncertain environments. Based on the above analysis, this article proposes the following research hypothesis:

Hypothesis 1. Relational embeddedness has an inverted U-shaped influence on dynamic capabilities.

### Relational Embeddedness and Ambidextrous Learning

To adapt to the particularity of the BOP market, the firm must shift their focus of competition from within-firm to learn through relationships. Improving one's resources and abilities through cooperative learning has become an important motivation for cooperative relations (Alatwi et al., 2021). March (1991) proposed that there are ambidextrous learning behavior of exploratory learning and exploitative learning in organizations. Compared with previous learning, ambidextrous learning considers the impact of the external environment, involving the organization's communication and interaction with external stakeholders and the environment. Specifically, exploratory learning refers to pursuing new knowledge, which is mainly manifested in the enthusiasm of the organization to actively search for and create new technologies, new strategies, and new opportunities (Noni and Apa, 2015). Exploitative learning refers to the in-depth exploration of existing knowledge, including the refining, selection, implementation, and reuse of knowledge, and applying it to organizational management (Lichtenthaler, 2009; Yuan et al., 2021).

Relational embeddedness provides a bridge for the knowledge transfer and information exchange of network entities, thereby providing a diversified knowledge base for ambidextrous learning (Wang and Hsu, 2014). The role of relational embeddedness in exploratory learning is mainly manifested in the establishment channels for obtaining heterogeneous resources, especially local social capital and tacit knowledge at the BOP beyond its previous scope of experience and activities. Since the knowledge and skills of the BOP area focus on the accumulation of complex knowledge such as planting techniques, traditional crafts, folk secret recipes, and experience know-how. Their transfer in market relations would be too risky or impossible due to difficulty to codify (Davidsson and Honig, 2003; Phadungkiati and Connell, 2014). The application of new knowledge to production has also been accelerated. Joint problem-solving encourages learning and imitation among actors, and thus new knowledge can be faster applied to the innovation activities in a new setting.

The effect of strong ties on exploitative learning is mainly manifested in its ability to deepen relevant knowledge. Exploitative learning provides deeper knowledge and more skillful competencies and further ensures high efficiency and implementation of product development (Atuahene-Gima and Murray, 2007) as well as the optimization of organizational processes (Li et al., 2013). In the process of joint problem-solving between companies with strong relationships, the required knowledge can be well-transferred. It is conducive to the effective integration of local resources with the resources and capabilities that enterprises have accumulated to improve technologies and models so that the value creation potential of obtained local resources can be released completely.

However, excessive relational embeddedness leads to negative effects. For example, social relationships based on trust make individuals more vulnerable to opportunistic actions, increase the cost of management and maintenance of the relationship, which do harm to the acquisition and application of innovative resources or information by the enterprises (Goel et al., 2005). Excessive information sharing leads to path dependence and organizational inertia. The lock-in effect causes redundancy of resources and information between each other, forming an inert relationship in knowledge acquisition, weakening the motivation of continuous learning, and inhibiting knowledge transfer (Villena et al., 2011). Meanwhile, knowledge is the key to a firm's ability to maintain its dynamic capabilities. Excessive reliance on joint problem-solving will ossify the firm's thinking and affect the acquisition and application of new knowledge. Based on the above analysis, the paper proposes the following research hypotheses:

Hypothesis 2. Relational embeddedness has an inverted *U*-shaped influence on exploratory learning.
Hypothesis 3. Relational embeddedness has an inverted *U*-shaped influence on exploitative learning.

### The Mediating Role of Ambidextrous Learning

Eriksson's (2014) research stated that organizational learning is one of the factors that promote the formation and improvement of dynamic capabilities. Exploratory learning has the characteristics of uncertain income and uncertain learning direction. Trust and information sharing mechanisms established by relational embeddedness facilitate jointly solving new problems, developing new technologies, and enhancing exploratory learning. This process increases the number and types of organizational knowledge reserves and enhances awareness of opportunities and crises (Lichtenthaler and Muethel, 2012; Nieves and Haller, 2014). Exploratory learning enables enterprises to get rid of the strong “rigidity” of existing strategies, technologies, and business processes formed in the aspects of existing experience and practices as well as overcome corporate inertia and path dependence, thereby adjusting respond to the external environment themselves promptly. Additionally, relational embeddedness can not only establish channels to obtain scarce resources but also poses opportunities for exploratory learning to develop a native capability suitable for the BOP market (Hart and London, 2005), and change organizational routines, improving environmental adaptation capability. Exploratory learning also affects the firm's ability to adapt to an environment full of uncertainties and the speed of decision-making. Extensive search and experimental learning behavior in communication and cooperation with other enterprises and institutions identify new technologies in the environment and create new opportunities to develop unique products, services, and business models (Prahalad, 2012; Zhao et al., 2020), improving environmental shaping capability. Joint problem-solving mechanism can also strengthen learning behavior in network relationships, permitting companies to adopt innovative thinking and adjust strategies to respond to market opportunities (Mcevily and Marcus, 2005).

Trust and cooperation enhance the willingness of organization members to exchange and absorb relevant knowledge as well as expand the content and depth of knowledge resources, promoting dynamic capabilities (Liao et al., 2009). The relevant resource accumulated in the BOP market helps to enrich knowledge and experience within the organization, enhancing the enterprises' capability to perceive opportunities and threats in the BOP market (Lichtenthaler and Muethel, 2012; Li and Lee, 2015). Through exploitative learning, enterprises update their knowledge and technology, which will help to understand market knowledge, market segmentation, and current forms of competition, and improve the ability of enterprises to obtain and utilize opportunities promptly. Enterprises can also change the competitive environment, create opportunities or avoid the risks of technological changes in the industry. Based on the above analysis, we propose the following research hypotheses:

Hypothesis 4. Exploratory learning mediates the relationship between relational embeddedness and dynamic capabilities.
Hypothesis 5. Exploitative learning mediates the relationship between relational embeddedness and dynamic capabilities.

### The Moderating Role of Prior Experience

As mentioned above, relational embeddedness facilitates enterprises building cooperative networks, where enterprises can actively acquire diversified information, and gain opportunities for new knowledge acquisition and application. We further discussed how the interaction between relational embeddedness and human capital affects ambidextrous learning. Knowledge can give it the potential to discover opportunities and new knowledge (Davidsson and Honig, 2003). As is well-known, knowledge can be got from formal education and experience. Prior experience refers to the knowledge, skills, and concepts accumulated in firms' business practices such as industry experience and target market experience. It can inform corporate decision-making in a volatile and changing environment, and effectively identify market opportunities (Cassar, 2014). The existing study confirms that the accumulation of prior experience is the basis for enterprise growth, and new enterprises with prior experience will have better performance expectations (Hopp and Sonderegger, 2015). We believe that prior experience is closely related to ambidextrous learning in the BOP context.

Some scholars have found that practitioners with more experience have more business awareness. This is a kind of tacit knowledge, which guides the sharing and exploration of new knowledge, accurately obtaining high-quality information. In addition, differences in prior experience largely affect the interpretation and understanding of the acquired information and knowledge (Shane and Venkataraman, 2000). According to the research of Garaud and Kumaraswam (2010), differences in prior experience will lead to different knowledge structures in enterprises, forming differences in organizational learning effects. Prior experience can also provide a basis for enterprises' decision to embed in the BOP market, and affects their understanding of the market and product development (Shane, 2000). Gregoire and Shepherd (2012) pointed out that prior experience enables deeper processing of information, identifying structural similarities between technical information and market needs, and then discovering more innovative and feasible opportunities. In short, prior experience promotes the acquisition and application of knowledge by enhancing the awareness and ability in the BOP market. Therefore, we propose the following hypotheses:

Hypothesis 6. The inverted *U*-shaped of relational embeddedness on exploratory learning increase with the increase of prior experience.
Hypothesis 7. The inverted *U*-shaped of relational embeddedness on exploitative learning increase with the increase of prior experience.

**Figure 1:**
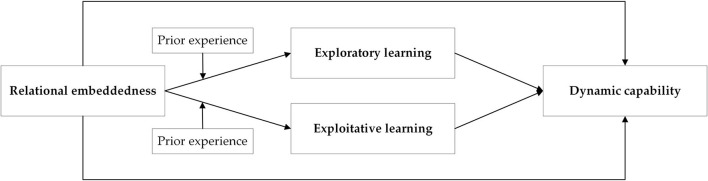
Theoretical research framework.

Figure 1 summarizes all the hypotheses and depicts our theoretical framework.

## Research Design

### Data Sources and Sample

To examine the impact of relational embeddedness on ambidextrous learning and BOP-oriented dynamic capabilities, it is necessary to collect data from enterprises embedded in the BOP market. This research finally selects enterprises in those industries located in concentrated contiguous areas in the Qinba Mountains as the main survey objects. Qinba Mountains is one of the concentrated contiguous destitute areas in China, with abundant and diverse resources, covering a large number of poor groups. The government has increased investment and support for this area, and a large number of advantageous industries have emerged, as well as many enterprises developed relying on local advantageous resources. This area is typical and representative.

This research employs a questionnaire survey method to collect data, which is divided into two stages: pre-research and formal research. The survey started on March 21, 2021, and ended on July 21, 2021, lasting for 4 months. The specific process is as follows: (1) Pre-research. To better fit the purpose of this research, we selected the mature scales from abroad according to the research goals and related theories. The researcher translated them into Chinese, and combined them with the existing domestic scale for semantic adjustment, forming the most primitive survey questionnaire. The questionnaire uses a Likert five-level scale for measurement, where 1 means strongly disagree and 5 means strongly agree. The initial phase of the pre-research took place in an academic setting, where the adjustments were made based on the feedback of 1 professor and 4 associate professors. There was a rewording of some questions to improve clarity, specificity, and brevity. Subsequently, the pre-research was conducted on 6 employees of BOP-oriented enterprises in Qinba Mountains from the same background as the target respondents. Then, some further refinements of the wording were performed to ensure the interviewees accurately understood them, fitting the BOP context. (2) Formal research. We distributed the survey and collected responses using a popular online platform (https://www.credamo.com/) with a total of over 2.8 million registered samples, comprehensive coverage of all provincial administrative regions in China, and support for hundreds of user tags. Based on offline interviews with local enterprises and the investigation through online channels such as government websites, we selected industries that absorb a large number of BOP groups, such as manufacturing, wholesale and retail, agriculture, biology and medicine, and transportation and logistics, etc. Before distributing the questionnaires, the sample characteristic values were set in the online system, such as industry, enterprise type, etc. Then, we used the electronic fence function of the website to set the area where the questionnaires will be delivered. We distributed 600 questionnaires online targeting enterprises located in concentrated contiguous areas in the Qinba Mountains Shaanxi province. To encourage responses, the survey offered some monetary rewards to respondents who completed it. The survey ran for more than 1 month and 315 respondents filled the questionnaire, with a return rate of 52.5%. We deleted those responses that were incomplete, finished too fast (e.g., in <3 min), or with wrong answers to test questions (with the given answer), or these incomplete or obvious problems (including opposite intentions, consistent answers, and obvious regularity). Furthermore, by calculating Cook's distance, we drew a scatterplot, identified the outliers and deleted them. A total of 51 were excluded. This process leaves us 264 useable responses, and the response rate of valid questionnaires is 44%.

Among the samples, females accounted for 58.33% and males accounted for 41.67%. Employees under the age of 20 accounted for 10.23%, 21–30 years old accounted for 32.58%, 31–40 years old accounted for 30.3%, and 40 years old above accounted for 26.89%. 34.85% of employees with a junior college degree and below, 54.55% of undergraduates, and 10.61% of masters and above. These respondents included grassroots staff (61.7%), managers (38.3%) in firms. These data suggest that the respondents were experienced and knowledgeable about the issues under study, which increased our confidence in the quality of the data. The sample covered a range of industries, including manufacturing (21.59%), wholesale and retail (18.18%), agriculture (37.88%), biology and medicine (16.29%), and transportation and logistics (6.06%).

### Measures

#### Independent Variables

The items of relational embeddedness are mainly designed from three aspects: mutual trust, information sharing, and joint problem-solving. This study is mainly based on the scale used by Mcevily and Marcus (2005) and Capaldo (2014) to measure the cooperative relationship mechanism. To this end, four items are used to measure mutual trust, four items are used to measure information sharing, and three items are measured to joint problem-solving.

#### Dependent Variables

According to the viewpoint of Teece (2007), dynamic capability is divided into three dimensions: sensing capability, adaptation capability, and shaping capability. Drawing lessons from research by Gibson and Birkinshaw (2004) and Wilden et al. (2013), four items are used to measure sensing capability, three items are used to measure adaptation capability, and three items are used to measure shaping capability.

#### Mediator

Ambidextrous learning is based on the research of Chung et al. (2015), adopting five items to measure exploratory learning and exploitative learning respectively.

#### Moderator

Based on previous research, prior experience measures whether a company has the knowledge, skills, and experience needed for a new market. Respondents provided whether their firms had experience in the industry or target market in which their new business competes. 1 means experience, 2 means no experience.

#### Control Variables

Firms of varying ages and sizes and in multiple industries present distinct ambidextrous learning (Jansen et al., 2009). Since firm age expresses a firm's development stage and is associated with its exploration and exploitation. As larger organizations may have more resources yet may lack the flexibility to achieve ambidextrous learning. Furthermore, industry effects may influence the extent to which organizations pursue exploratory and exploitative learning. Following Jansen et al. (2009), we select firm age, size, and industry as control variables in this study. Among them, the firm age is measured by the natural logarithm of the company's establishment years. The firm size is measured by the natural logarithm of the number of employees to avoid excessive values. The industry to which the company belongs the agriculture, forestry, animal husbandry, and fishery industries are set to 1, and the other industries are set to 2.

## Results

### Reliability and Validity Test

SPSS25.0 software was used to analyze the reliability of relational embeddedness, exploratory learning, and dynamic capability scales. Among them, the Cronbach's α coefficients of relational embeddedness, exploratory learning, exploitative learning, and dynamic capabilities are 0.941, 0.877, 0.927, and 0.952, respectively, which are all >0.800, indicating good reliability. It shows that the scales of relational embeddedness, ambidextrous learning, and dynamic capabilities have good reliability, consistency, and stability. At the same time, the CITC values of the overall correlation coefficients of the scale items were all greater than the acceptable standard of 0.40, and the value of α did not increase when any item was deleted. Therefore, the overall reliability of the scale is relatively high.

The validity test was carried out from two aspects of construct validity and convergence validity. First of all, all scale items in this study were sourced from mature research scales at home and abroad and discussed with experts in related fields before the formal survey. After small pre-research, some inappropriate items were modified. Therefore, the scale of this study has high content validity. Moreover, after exploratory factor analysis, it is found that the KMO value is 0.959, and the Chi-square value of Bartlett's test is 6635.818. We employed AMOS 24.0 software to perform confirmatory factor analysis on the variables selected in this study to test the aggregate validity of relational embeddedness, exploratory learning, exploitative learning, and dynamic capabilities. The confirmatory factor analysis results showed that χ^2^/df = 1.764, <3, RMSEA = 0.054, <0.08, CFI = 0.953, >0.9, AVE value >0.5, combined reliability CR >0.7, indicating variables' reliability of the scales is ideal.

Secondly, as shown in Table 1, the factor loadings of all items were larger than 0.7; thus, the convergent validity of each item was good. Table 2 shows that the square roots of the AVE for each variable were greater than the Pearson correlation coefficients, so the questionnaire in this study has a good degree of discriminate validity, indicating that the validity of the measurement was good. No measurement error correction for independent or dependent variables and range restriction correction were performed in the paper.

**Table 1:** The test results for variable reliability.

**Constructs/measurement items**	**Standardized factor loadings**	**CR**	**AVE**	**Cronbach's α**
Relational embeddedness (RE)		0.943	0.620	0.941
1 Partners can seek truth from facts when negotiating	0.708			
2 Partners can keep their promises in cooperation	0.734			
3 Partners do not mislead the company.	0.63			
4 Partners will not use the companies' weaknesses to obtain improper benefits	0.667			
5 Partners exchange information with the companies frequently, not limited to established agreements	0.799			
6 Partners and the companies remind each other of possible problems and changes	0.79			
7 Partners and companies provide each other with the required information as much as possible	0.855			
8 Partners share future development plans with the companies	0.775			
9 Partners and companies can be jointly responsible for completing tasks	0.849			
10 Partners and companies can help each other to solve the problems encountered in cooperation	0.851			
11 Partners and companies work together to overcome difficulties	0.851			
Exploratory learning (ER)		0.876	0.585	0.876
1 Attaches importance to acquiring product/service strategic knowledge that involves trials and high market risks in the BOP market	0.757			
2 Extensively searches for information in the BOP market to ensure trials in the development of new products/services	0.739			
3 For the purpose of acquiring new knowledge and developing products/services in the BOP market (e.g., new markets and technical experience)	0.771			
4 Collects new information beyond the existing technical experience in the BOP market	0.745			
5 Collects new information on the BOP market and learn new knowledge in the development of new products/services	0.811			
Exploitative learning (EI)		0.927	0.718	0.927
1 Searches for information and improve methods and ideas for solving problems in the development of new products/services	0.848			
2 Search the BOP market for ideas and information that can guarantee production capacity	0.846			
3 Finds common and widely accepted methods and paths in the BOP market to solve problems in product development/service development	0.895			
4 Uses methods of collecting information (for example, survey current customers and competitors) to understand and update the company's current product/service market experience	0.820			
5 Emphasizes the use of knowledge related to existing product/service experience	0.824			
Dynamic capability (DC)		0.951	0.662	0.951
1 Focuses on best practices within the company	0.844			
2 Collects economic information related to operations and operating environment	0.815			
3 Uses existing processes to identify BOP market segmentation and changes in consumer demand	0.820			
4 Encourages employees to participate in the activities of industry associations	0.822			
5 Encourages employees to challenge outdated traditions and practices	0.791			
6 Quickly responds to changes in the BOP market	0.823			
7 Updates the management process according to the priority of BOP market business development	0.818			
8 Frequently adopts new management methods	0.811			
9 Frequently adopts new or substantively changed marketing methods or strategies	0.825			
10 Frequently adopts new or substantive changes in business processes	0.766			

**Table 2:** Descriptive statistics for the main variables.

	**Age**	**Size**	**Industry**	**RE**	**ER**	**EI**	**DC**	**Experience**
Firm age								
Firm size	0.504**							
Industry	−0.192**	−0.099						
RE	0.219**	0.052	−0.209**	0.787				
ER	0.191**	0.094	−0.028	0.711**	0.765			
EI	0.205**	0.048	−0.092	0.718**	0.646**	0.847		
DC	0.050	0.051	−0.011	0.552**	0.496**	0.487**	0.814	
Experience	0.223**	0.013	−0.149*	0.522**	0.363**	0.475**	0.273**	

The diagonal values (in bold) are the square roots of AVE, others are Pearson correlations. *p < 0.05, **p < 0.01; two-tailed test.

### Descriptive Statistics and Correlation Analysis

It can be seen from Table 1 that the average values and standard deviations of the variables are within the normal range. The independent variable (relational embeddedness), the dependent variable (dynamic capability), and the mediating variables (exploratory learning, exploitative learning) all show a strong correlation. They have a significant positive correlation with the dependent variable. The main effect of this study has been initially verified, but further analysis is needed with multiple regression.

### Regression Analysis and Hypothesis Testing

Based on the preliminary verification of research hypotheses by correlation analysis, this paper uses hierarchical regression analysis to explore the impact of relational embeddedness and ambidextrous learning on dynamic capabilities. The first layer puts the firm age, firm size, and industry to which it belongs as control variables. The second layer inputs independent variables and their square terms based on the research hypothesis model. The third layer inputs mediating variables (exploratory learning, exploitative learning). The fourth layer inputs the moderating variables (prior experience), and the interaction terms with independent variables and their square terms. Before calculating the square or interaction terms, the relevant variables are mean-centered to reduce the impact of multi-collinearity. After centralized processing, the VIF value is between 1 and 3, indicating that the collinearity problem will not affect the analysis results.

#### The Main Effect of Relational Embeddedness on Dynamic Capabilities

Regression analysis was performed by SPSS 25.0 software, and the test results are shown in Table 3. Model 1 examined the influence of control variables on the dynamic capabilities of the firms. Based on Model 1, the relational embeddedness variable and its square term were added to build Model 2, and the result shows that they have a significant impact on the dynamic capabilities (β_1_ = −0.365^***^, *P* < 0.001; β_2_ = 0.615^***^, *P* < 0.001), H1 was supported. Considering the control variables, the explanation rate of the relational embeddedness to the variance of dynamic capabilities reached 45.1%, indicating that the data fit the model well. Relational embeddedness and dynamic capabilities are in an inverted *U*-shaped relationship.

**Table 3:** Regression results of direct effect.

**Variables**	**Dynamic capabilities**			
	**Model 1**	**Model 2**	**Model 3**	**Model 4**
Firm age	−0.038	−0.054	−0.063	−0.084
Firm size	0.021	0.080	0.042	0.072
Industry	−0.002	0.059	−0.014	0.027
RE		0.615^***^		
RE square		−0.365^***^		
ER			0.503^***^	
EI				0.503^***^
*R* ^2^	0.003	0.451	0.248	0.244
Adj *R*^2^	−0.008	0.441	0.237	0.233
*F*	0.297	42.423	21.388	20.922

*** *p < 0.001*.

#### The Mediating Effect of Ambidextrous Learning

Hierarchical regression was used to analyze the mediating effect of ambidextrous learning between relational embeddedness and dynamic capabilities. The regression results are shown in Table 4. It can be seen from Model 5 that relational embeddedness has a significant positive impact on exploratory learning, and its square term negatively affects exploratory learning significantly (β_1_ = −0.095^*^, *P* < 0.05; β^2^ = 0.725^***^, *P* < 0.001), indicating that there is an inverted *U*-shaped relationship. H2 was supported. Model 3 shows that exploratory learning positively affects dynamic capabilities. Model 6 incorporates exploratory learning into the regression equation, and the results show that exploratory learning significantly promotes enterprises' dynamic capabilities (β_3_ = 0.131^*^, *P* < 0.05). The influence of the primary term of the relational embeddedness on the dynamic capabilities is significant, and the influence of its square term on the dynamic capabilities is weakened (β_1_ = −0.352^***^, *P* < 0.001), indicating that exploratory learning mediates the relationship between relational embeddedness and dynamic capabilities. H4 was supported. Following models 7 and 8 successively verify the mediating effect of exploitative learning in relational embeddedness that affects the dynamic capabilities. Specifically, model 7 shows that relational embeddedness has a significant positive impact on exploitative learning, and the square term regression coefficient is significantly negative (β_1_ = −0.104^*^, *P* < 0.05; β_2_ = 0.725^***^, *P* < 0.001), indicating that there is an inverted *U*-shaped relationship. Therefore, H3 was supported. Model 4 shows that exploitative learning positively affects dynamic capabilities. Model 8 indicates that after controlling for exploitative learning variables, the regression coefficient of the square term is significantly negative. Compared with the effect on dynamic capabilities alone, the absolute value of the coefficient was reduced, and the regression coefficient of exploitative learning is significantly positive, indicating that exploratory learning plays a significant mediating role in the inverted *U*-shaped relationship. Therefore, H5 was supported.

**Table 4:** Results of mediation regression analysis and moderated regression analysis.

**Variables**	**ER**	**DC**	**EI**	**DC**	**ER**	**EI**
	**Model 5**	**Model 6**	**Model 7**	**Model 8**	**Model 9**	**Model 10**
Firm age	0.041	−0.059	0.083	−0.063	0.037	0.071
Firm size	0.041	0.075	−0.018	0.082	0.038	−0.022
Industry	0.139^**^	0.040	0.057	0.052	0.124^**^	0.027^**^
RE	0.725^***^	0.521^***^	0.725^***^	0.534^***^	0.919^***^	0.204^***^
RE square	−0.095^*^	−0.352^***^	−0.104^*^	−0.353^***^	−0.231	−0.375^**^
ER		0.131^*^				
EI				0.112^†^		
EXP					−0.008	−0.011
RE*EXP					−0.207	−0.510^***^
RE square *EXP					0.148	0.298^*^
*R* ^2^	0.516	0.459	0.533	0.457	0.522	0.566
Adj *R*^2^	0.506	0.447	0.524	0.444	0.507	0.552
*F*	54.984	34.409	58.974	36.063	34.800	41.514

† *p < 0.1;*

* *p < 0.05;*

** *p < 0.01;*

*** *p < 0.001*.

#### The Moderating Effect of Prior Experience

Introducing the interaction terms of the independent variable and its square term and moderating variables, the study tested the moderating effects of prior experience (models 9 and 10). The results are shown in Table 4.

Models 9 and 10 show the moderating effect of prior experience. The results show that the coefficient of the relational embeddedness and its square term and the interaction terms of the prior experience are significant (β = 0.298^*^, *P* < 0.05) between relational embeddedness and exploitative learning, that is, the moderating effect of prior experience does exist, and H7 was supported. As for exploratory learning, the interaction coefficients between relational embeddedness and its square term and prior experience are not significant. It can be seen that there is no moderating effect between relational embeddedness and exploratory learning. H6 was not supported.

**Figure 2:**
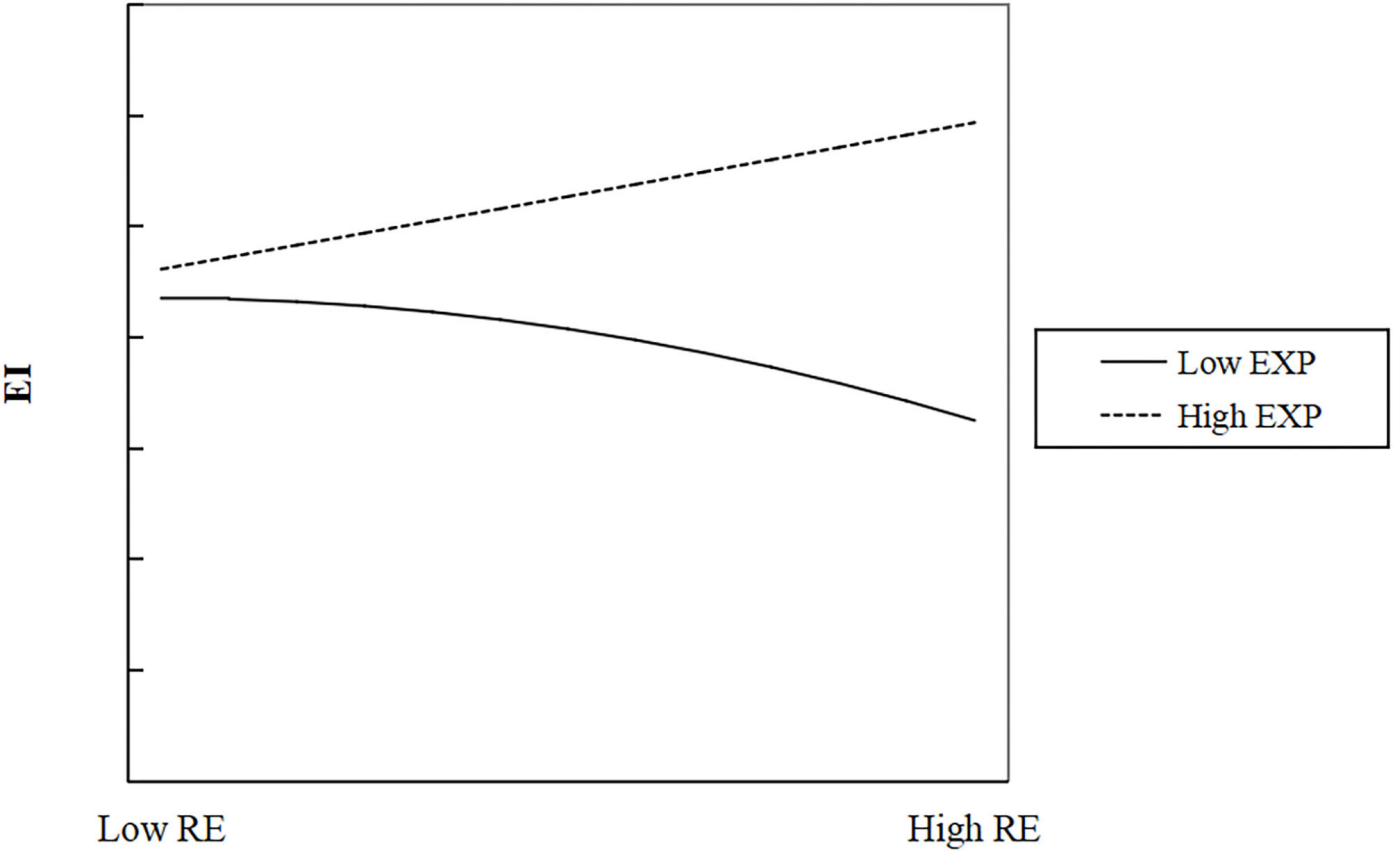
The moderating effect of prior experience between relational embeddedness and exploitative learning.

To intuitively reflect the moderating effect of prior experience between relational embeddedness and ambidextrous learning, we draw a schematic diagram of moderating effect according to the method recommended by Cohen (2003). When drawing the graph, the mean plus or minus one standard deviation is used to indicate the level of relational embeddedness and prior experience, as shown in Figure 2.

Figure 2 shows that the non-linear relationship between relational embeddedness and exploitative learning moves with changes in prior experience. Prior experience strengthens the positive influence of relational embeddedness on exploitative learning and moves to the upper right while no-prior experience strengthens the negative influence of relational embeddedness on exploitative learning and moves to the lower left. It shows that prior experience plays a positive moderating role, so H7 was supported.

## Discussion

In this paper, using survey data from 264 valid samples, we examined how relational embeddedness affects dynamic capabilities *via* ambidextrous learning. The study found that relational embeddedness has a curvilinear effect on dynamic capabilities, as well as ambidextrous learning. On this basis, we found that the relationship between relational embeddedness and dynamic capabilities is positively mediated by ambidextrous learning. Furthermore, the study also found that prior experience positively moderates the relationship between relational embeddedness and exploitative learning.

### Theoretical Implications

The current study has made several theoretical implications. First, this study provides a new understanding to reveal the effect of relational embeddedness and extends its application to dynamic capabilities in the BOP context. Whereas a plethora of studies has claimed conceptually that relational embeddedness practices influence firms' dynamic capabilities (e.g., Frasquet et al., 2018; Alinaghian et al., 2020), our study contributes to the dynamic capabilities literature by empirically supporting the notion that relational embeddedness is an enabler of dynamic capabilities. However, we found that the impact of relational embeddedness on dynamic capabilities is not just a simple linear relationship. After a certain boundary point, relational embeddedness has a decreasing effect on dynamic capabilities. That is, higher relational embeddedness is not necessarily better (Burt, 1992; Uzzi, 1997; Obukhova and Zhang, 2017). This is inconsistent with Rodrigo-Alarcón et al. (2018), Ai and Peng (2021), Zheng (2021), and Zhou et al. (2021), who found that relational capital may positively predict dynamic capabilities. As mentioned earlier, most of the previous literature shows the positive effects of relational embeddedness. The negative effects (so-called actors' social overembeddedness) are rarely the subject of in-depth considerations (Mitrega and Zolkiewski, 2012; Obukhova and Zhang, 2017). However, Czernek-Marszaek (2020b) confirmed that social embeddedness has not only positive but also negative effects on economic activity, such as high maintenance cost, redundant information, opportunistic behaviors, and lower adaptation abilities caused by adjusting to known partners. These negative effects will inhibit the dynamic capabilities of enterprises. Our findings empirically reveal that growing partnerships are a double-edged sword. That is, moderate partnerships of enterprises can bring advantages while too close partnerships lead to disadvantages. This confirms the view that an enterprise that is too deeply socially embedded is less adaptable (Nahapiet and Ghoshal, 1998; Mizruchi and Stearns, 2006). The study discloses an in-depth understanding of the process of the “embeddedness paradox” and facilitates understanding the antecedents of dynamic capabilities in the BOP context.

Second, the current study advances the literature of dynamic capabilities by showing that as a mediation device, ambidextrous learning may transform useable resources (source from relational embeddedness) into capability or advantages. In establishing this, it draws on the “resource-capability-high-order capability” framework (Winter, 2003; Cepeda and Vera, 2007; Wang and Ahmed, 2007), and builds on the basis of significant direct effects of relational embeddedness on ambidextrous learning as well as ambidextrous learning on dynamic capabilities. The research has found that moderate relational embeddedness enables enterprises to obtain key resources from bridged individuals or organizations, effectively absorb and transform the acquired more information and technologies, and enhance the dynamic capabilities of enterprises. The study indicates that exploratory learning and exploitative learning may enable firms to benefit from relational embeddedness. As stated by Wang et al. (2020), relational embeddedness improves the quality and quantity of knowledge acquisition and exchange, thereby helping enterprises explore and exploit knowledge from their environments. Some scholars claimed that ambidextrous learning is a necessary condition for the realization of dynamic capabilities (O'Reilly and Tushman, 2008; Yuan et al., 2021). Ambidextrous learning connects relational embeddedness with dynamic capabilities, through which available and new resources or knowledge can be used to improve functions of sensing, adaptation, and shaping the environment. Nevertheless, we found that excessive relational embeddedness will cause negative effects such as information redundancy, cognitive bias, and opportunistic behaviors, which restrains exploratory learning and exploitative learning, thereby inhibiting the dynamic capabilities. Therefore, enterprises need to continuously improve embeddedness strategies to provide sufficient relevant information and resources for ambidextrous learning, thereby improving dynamic capabilities. In a word, this is a supplement to the previous view and consolidated support for the dynamic capabilities of social capital through the perspective of organizational learning (Aranda et al., 2017).

Third, from the perspective of human capital, we explore the boundary problem of the role of relational embeddedness on the inverted *U*-shaped effect of exploratory learning and exploitative learning. It provides a new perspective for exploring the relationship between social capital, human capital, and ambidextrous learning. That is to say, relational embeddedness provides enterprises with more ways to acquire information and resources, but they are complementary to human capital. The resources needed for exploratory learning and exploitative learning come from social capital as well as human capital such as prior experience. Although some studies believe that prior experience will also bring disadvantages, such as cognitive inertia (overconfidence, minority principle), risk aversion, and lock-in effects (Simon et al., 2000), our research finds that prior experience positively moderates the relationship between relational embeddedness and exploitative learning in the BOP market, this is consistent with Hatch and Dyer (2004) and Politis (2005). They hold that the accumulation of prior experience is more likely to lead organizations to carry out exploitative learning behavior. The reason is that, due to the particularity of the BOP market, the prior experience can better identify the value of local resources, acquire tacit knowledge of the existing market, and promote experiential learning and transformation of existing knowledge. However, its moderating effect on exploratory learning was not significant. It may be because exploratory learning specifically refers to searching for experience and knowledge unrelated to the current experience to actively conduct experimental attempts. For example, in the BOP market, firms establish relationships with universities and research institutes to carry out project cooperation (breeding pig research and development, Drug Discovery, etc.), and these experiments are less affected by prior experience. This research inspires those enterprises should focus on summarizing successes and failures or bring in experienced teams to deal with the negative effects of overembeddedness, which will improve future exploitative learning outcomes. At the same time, it also enlightens us that exploratory learning and exploitative learning are related to social capital and human capital. The enterprises with different human capital may differ in the performance of relational embeddedness on ambidextrous learning. The study promotes understanding of the contextual factors that affect the inverted *U*-shaped relationship between relational embeddedness and ambidextrous learning and its contingency mechanism.

### Practical Implications

The research conclusions of this paper have important management enlightenment for BOP market-oriented enterprises.

(1) The research has practical enlightenment for improving the dynamic capabilities of enterprises. Actively maintaining appropriate contact with partners and cultivating external relationship networks increase opportunities for enterprises to obtain and use external resources that can enhance their dynamic capabilities. Studies have shown that excessive embeddedness can damage the dynamic capabilities of enterprises. Managers can't just rely on relational embeddedness as an informal network governance mechanism. In business practice, they should actively explore the combination of formal network governance mechanisms and informal network governance mechanisms to establish moderate relational embeddedness.
(2) Take note of the construction and cultivation of exploratory learning and exploitative learning. Ambidextrous learning is conducive to the improvement of organizational dynamic capabilities. The influence of relational embeddedness on dynamic capabilities is also partly realized through exploratory learning and exploitative learning, which emphasizes the importance of ambidextrous learning to improve dynamic capabilities. For enterprises in real situations, learning behavior is very important. In practice, enterprises should continue to expand the existing resource base and improve the diversity of capabilities through experiments, innovations, and other behaviors, to continue to gain competitive advantages in the ever-changing environment.
(3)Entering a new market is a process of learning by experiment. Establishing a higher level of local relational embeddedness do benefit from different resources and information. More important is how to use these resources and information. Enterprises should appropriately emphasize the effects of prior experience. Let them participate in the activities of enterprises embedding in the BOP market so that enterprises have the consciousness, ability, and opportunity to make full use of social capital, adjust or strengthen their networks to adapt to the environment, and improve future beneficial results.

### Limitations and Future Research

The research has the following limitations. First, in the process of data collection, although the universality and completeness of the data have been ensured as much as possible, the sample inevitably has limitations. In the future, the sample size and diversity should be expanded to further enhance the universality of the theory. Second, neither overembeddedness nor so-called underembeddedness is beneficial for a company. The question of what the optimal combination of strong and weak ties is, however, is still open. Third, the enterprises' practice generally includes exploratory learning and exploitative learning. In future research, more attention should be paid to practical significance, and the relative balance and interactive effects of exploratory learning and exploitative learning should be studied, rather than taking exploratory learning and exploitative learning as independent elements that have no mutual influence and relevance. Finally, the relationship between relational embeddedness and dynamic capabilities is very complex, and the perspectives and research entry points are also diverse. In the future, we can research more perspectives to finally improve our understanding of relational embeddedness, dynamic capabilities, and their relationship.

## Conclusion

Based on the literature review and hypotheses development, the study explores the influence of relational embeddedness on the dynamic capabilities *via* ambidextrous learning in the BOP context, as well as the moderating effect of prior experience. The results indicated that the impact of relational embeddedness on dynamic capabilities is a curvilinear relationship. Moderate relational embeddedness is an enabler of dynamic capabilities, but after a certain boundary point, overembeddedness has a decreasing effect on dynamic capabilities. Meanwhile, the positive or negative effects of relational embeddedness on dynamic capabilities are partially mediated by exploratory learning and exploitative learning. Finally, this study examined the contextual effect of prior experience on ambidextrous learning. Prior experience amplified the positive effect of relational embeddedness on exploitative learning and mitigated its negative effect. However, its moderating effect on exploratory learning was not significant. In conclusion, this research enriches our understanding of the dynamic capabilities' antecedents and the black box of the “embeddedness paradox” in the BOP context. We hope that our theoretical model and empirical evidence will inspire more attention to the potential mechanism between relational embeddedness and dynamic capabilities.

## Data Availability Statement

The raw data supporting the conclusions of this article will be made available by the authors, without undue reservation.

## Ethics Statement

Ethical review and approval were not required for the study on human participants in accordance with the local legislation and institutional requirements. Written informed consent for participation was not required for this study in accordance with the national legislation and the institutional requirements.

## Author Contributions

YZ designed and executed the study, analyzed the data, and prepared the first draft. JL planned and designed the study. WZ designed the study and reviewed and revised the draft. All authors read and approved the submitted version.

## Funding

This work was funded by the Department of Science and Technology of Shaanxi Province and Shaanxi Association for Science and Technology, Grant Number E219060016.

## Conflict of Interest

The authors declare that the research was conducted in the absence of any commercial or financial relationships that could be construed as a potential conflict of interest.

## Publisher's Note

All claims expressed in this article are solely those of the authors and do not necessarily represent those of their affiliated organizations, or those of the publisher, the editors and the reviewers. Any product that may be evaluated in this article, or claim that may be made by its manufacturer, is not guaranteed or endorsed by the publisher.
